# CDK2/9 blockade remodels chromatin and drives phenotypic plasticity in glioblastoma organoids

**DOI:** 10.1186/s12964-026-03272-6

**Published:** 2026-10-02

**Authors:** Philipp Kaps, Anne Sophie Hieltscher, Andrea Auditore, Christoph Kamper, Sophie-Charlotte Nelz, Jacqueline Heskamp, Paul Meißner, Annabell Wolff, Katharina Richter, Jakob Brandstetter, Piotr Grabarczyk, Sascha Troschke-Meurer, Burkhard Hinz, Felix Wittig, Daniel Dubinski, Florian Gessler, Thomas M. Freiman, Susanne Stählke, Philipp-Kjell Ficht, Lars Boeckmann, Christian Polley, Carl-Friedrich Classen, Christian Junghanss, Claudia Maletzki

**Affiliations:** 1 Department of Internal Medicine — Clinic and Polyclinic for Hematology, Hemostaseology, Oncology, Stem Cell Therapy and Palliative Medicine, Rostock University Medical Center, University of Rostock, Ernst-Heydemann-Straße 6, Rostock, D-18057 Germany; 2 Fraunhofer Institute for Cell Therapy and Immunology IZI, Department of Extracorporeal Therapy Systems, Rostock, Germany; 3 Department of Internal Medicine, Clinic of Nephrology, Rostock University Medical Center, University of Rostock, Rostock, Germany; 4 Department of Otorhinolaryngology, Head and Neck Surgery ‘’Otto Koerner‘’, Rostock University Medical Center, Rostock, Germany; 5 Department of Internal Medicine, Clinic III-Hematology, Oncology, University Medicine Greifswald, Greifswald, Germany; 6 Department of Pediatric Oncology and Hematology, University Medicine Greifswald, Greifswald, Germany; 7 Institute of Pharmacology and Toxicology, Rostock University Medical Center, Rostock, Germany; 8 Department of Neurosurgery, Rostock University Medical Center, University of Rostock, Rostock, Germany; 9 Institute of Cell Biology, Rostock University Medical Center, University of Rostock, Rostock, Germany; 10 Clinic and Policlinic for Dermatology and Venereology, University Medical Center Rostock, Rostock, Germany; 11 Faculty of Mechanical Engineering and Marine Technology, University of Rostock, Rostock, Germany; 12 University Children’s Hospital, Rostock University Medical Center, University of Rostock, Rostock, Germany

**Keywords:** DNA damage, Metabolic rewiring, Long non-coding RNA, Microglia, Intercellular crosstalk

## Abstract

**Background:**

Glioblastoma (GBM) remains a highly lethal brain tumor with limited therapeutic options and pervasive resistance to standard treatments. Cyclin-dependent kinases (CDKs), key regulators of cell cycle progression and transcription, are frequently dysregulated in GBM and contribute to tumor pathogenesis.

**Methods:**

We employed a range of preclinical GBM models—including 2D cultures, 3D spheroids, patient-derived organoids (PDOs), and multicellular 3D bioprints—to evaluate the effects of the CDK2/9 inhibitor fadraciclib in comparison to the standard-of-care temozolomide. Endpoints included cell viability, stress responses, immune-modulatory effects, and transcriptomic reprogramming.

**Results:**

Fadraciclib robustly reduces cell viability and invasion in all patient-specific 2D- and 3D models. This effect was independent of O6-methylguanine-DNA methyltransferase (MGMT) promoter methylation status, demonstrating superior efficacy to temozolomide. Mechanistically, fadraciclib disrupted lysosomal, mitochondrial, and endoplasmic reticulum integrity and increased γH2AX levels, indicating enhanced DNA double-strand breaks. Seahorse analysis demonstrated impaired mitochondrial bioenergetics following fadraciclib treatment. In a triple-culture blood-brain barrier (BBB) model, fadraciclib demonstrated effective BBB penetration and antitumoral activity against GBM cells, while exhibiting minor cytotoxicity toward brain-specific normal cells. In semi-autologous co-culture systems, fadraciclib inhibited GBM invasion, promoted intercellular communication via Connexin 43 upregulation in microglia, and activated peripheral T cells. Transcriptomic analyses of treated patient-derived organoids revealed widespread transcriptional reprogramming, including downregulation of oncogenic long non-coding RNAs (*MALAT1*,* POM121L9*), suppression of immune-related pathways, and upregulation of the tumor suppressor *LZTS1*. Increased histone-associated gene expression suggests chromatin remodeling and induction of a quiescent tumor cell state. Subtype analysis indicated a shift toward a proneural molecular signature. Notably, the antitumoral effects of fadraciclib were preserved in multicellular biomimetic bioprints, whereas nonmalignant astrocytes and microglia were not affected. Therapeutic activity also coincided with altered secretion profiles of extracellular vesicles (EV). EVs from reactive astrocytes contained higher amounts of Fas, which may act as modulator of neuroinflammation.

**Conclusion:**

CDK2/9 inhibition shows promise as a therapeutic strategy for GBM. Fadraciclib exhibits multifaceted anti-tumor activity and spares nonmalignant cells of the central nervous system, supporting its further development in therapies that target transcriptional and immune pathways, as well as quiescent tumor cell populations.

**Graphical Abstract:**

**Figure:**
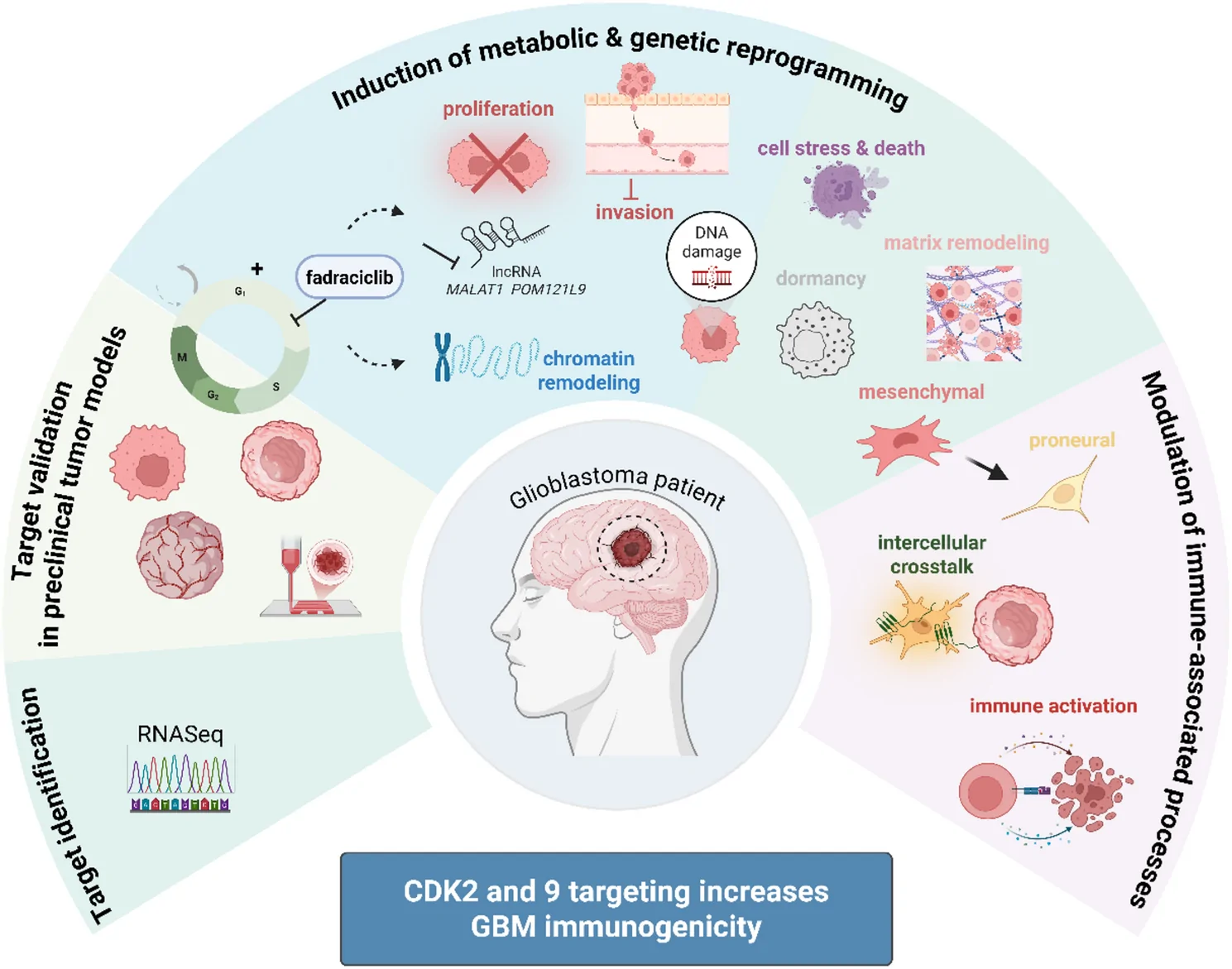

**Supplementary Information:**

The online version contains supplementary material available at https://doi.org/10.1186/s12964-026-03272-6 .

## Introduction

Central nervous system tumors are among the most lethal cancers, with IDH-wildtype glioblastoma (GBM)—a WHO grade 4 tumor—being the most common malignant subtype [1]. Despite therapeutic advances, GBM remains largely incurable, with a five-year survival rate below 10% [2]. Genetic alterations and associated druggable targets are rare in GBM, but accumulating evidence points to the critical role of cell cycle dysregulation in disease progression [3, 4].

Aberrant cell cycle regulation is a key driver of uncontrolled proliferation and tumorigenesis. This includes overexpression of cyclin-dependent kinases (CDKs), upregulation of cyclins, or loss of CDK inhibitors (CDKis) [4–6]. Notably, nearly half of all brain tumors harbor genetic alterations in cell cycle regulatory pathways [7], and in previous work, we demonstrated that pharmacologic inhibition of CDK4/6 or CDK7 reduced GBM cell viability, supporting the rationale for CDK-targeted therapies [8–10]. Recent data support their use as microenvironmental-targeted therapies, potentially in combination with repurposed letrozole [11].

Fadraciclib is a second-generation, selective inhibitor of CDK2/CDK9 [12], currently under clinical investigation for various hematologic and solid malignancies, including GBM (ClinicalTrials.gov ID: *NCT04983810*, registration date: 30.07.2021). Its dual CDK2/9 inhibition targets key pathways in cell cycle progression and transcriptional regulation. CDK2 promotes G1/S phase transition and DNA replication through interactions with cyclins E/A, and its elevated expression in microvascular proliferation regions has recently been linked to GBM risk [13–15]. CDK9, as part of the positive transcription elongation factor b (P-TEFb) complex with cyclin T1, interacts with RNA polymerase II (RNAPII) [16, 17] and initiates phosphorylation of transcriptional regulatory proteins [18]. While CDK9 expression alone does not correlate with GBM prognosis [19], its role in modulating DNA repair and metabolic stress increases tumor vulnerability to treatment, highlighting CDK9 as a promising therapeutic target in GBM [20–22].

Recent developments in preclinical and translational research have shifted from classical in vivo models to sophisticated in vitro approaches, collectively referred to as new approach methodologies. Among them, patient-derived organoids (PDOs) are increasingly employed as clinical decision-support tools, capturing genetic and nongenetic determinants of drug response [23–25]. They enable rapid testing of single drugs and combinations, reveal unexpected vulnerabilities, and have guided personalized treatments across multiple cancer types, including refractory cases. These factors lay the groundwork for preclinical evaluation of fadraciclib in GBM. Still, empirical evidence of its antitumor efficacy in GBM remains limited. Dual CDK2/9 inhibition disrupts oncogenic signaling and transcriptional survival programs while enhancing antitumor immunity, yet its molecular and immunological mechanisms in the GBM microenvironment are poorly understood. Here, we systematically examine CDK2/9 inhibition across preclinical GBM models and its impact on immunological characteristics.

## Materials and methods

### Cell culture

Patient-derived GBM cell lines were established from WHO grade 4 patients (Table 1). Samples were obtained from the Department of Neurosurgery, Rostock University Medical Center (Germany) with informed consent and approval from the local Ethics Committee (A2018-0167), in accordance with accepted guidelines for human materials.

**Table 1:** Clinical data of GBM cases included in this study

Patient ID	Gender/age	Tumor localization	MGMT status	Mutations
GBM03	m/81	right temporal lobe	unmethylated	*TP53* (R273H (VAF: 48.6%); R248Q (VAF: 50.3%); P72R (VAF: 99.6%); *KDR* Q472H (benign, VAF: 35.0%)
GBM06	m/71	temporal lobe	methylated	*TP53* G244A (VAF: 99.0%); P72R (VAF: 100.0%); *PIK3CA* I391M (VAF: 30.0%)
GBM11	m/73	left frontal lobe	methylated	N/A
GBM14	m/63	left temporal lobe	unmethylated	*TP53* V173L (VAF: 58.6%); P72R (VAF: 58.3%); *KDR* Q472H (VAF: 54.0%); *PTEN* D252Y (VAF: 100.0%)
GBM15	m/40	left parietal lobe	unmethylated	*TP53* P72R (VAF: 99.0%)
GBM23	m/73	left temporofrontal lobe	methylated	N/A
GBM26	f/73	right occipito-temporal lobe	methylated	*ATM* R2443* (VAF: 51.6%)
GBM31	f/61	right frontal lobe	unmethylated	N/A
GBM37	f/74	N/A	methylated	N/A
GBM39	m/58	left temporal lobe	unmethylated	*TP53* R342* (VAF: 81.2%)
GBM40	m/68	left parietal lobe	unmethylated	N/A
GBM41	m/59	right frontal lobe	unmethylated	*PIK3CA* E545K (VAF: 50%), *PTEN* M134V (VAF: 56.7%), *TP53* C135R (VAF: 64%);
GBM44	m/83	left parietooccipital lobe	methylated	*TP53* R282W (VAF: 87%)

All specimens were diagnosed as WHO grade 4 GBM with wild-type IDH status

*Abbreviations*: *MGMT* O6-methylguanine-DNA methyltransferase, *m* male, *f* female, *N/A* Not analyzed

2D-cultured GBM cells were maintained in DMEM/F12 (+ 10% fetal calf serum, 6mmol/l L-glutamine, and 1% penicillin/streptomycin (PAN-Biotech, Germany)). Human astrocytes (hAstro; ScienCell, USA) were grown on poly-L-lysine-coated vessels in supplemented astrocyte medium. hMC3 cells (ATCC CRL-3304™, USA) were maintained in EMEM. All cell lines were incubated at 37 °C with 5% CO₂ in a humidified atmosphere.

### Concentration-response curve

GBM cells were seeded in 96-well plates (5,000 cells/well), incubated for 24 h, and treated with increasing concentrations of fadraciclib for 2 × 72 h; controls received medium only. In selected experiments, temozolomide (10 µM) was added. Fadraciclib and temozolomide were dissolved in DMSO. The final DMSO concentration in the incubation medium was 0.1% (v/v) in both compound-treated and vehicle control groups. Cell viability was determined by crystal violet staining (0.2%, 10 min). Absorbance was measured after 1% SDS addition at 570 nm using a Tecan Infinite 200 Pro plate reader (TECAN, Männedorf, Switzerland).

### Evaluation of blood-brain barrier (BBB) permeability

The human induced pluripotent stem cell (hiPSC)-derived triple-culture model resembling the BBB was assembled as previously described with minor modifications [26]. Briefly, apical and basal side of the TC-inserts (growth area: 0.33 cm^2^, pore size: 0.4 μm, transparent; Sarstedt, Nümbrecht, Germany) were coated with 200 µg/ml Matrigel GFR (Corning, Corning, NY, USA) for 1 h at room temperature. Astrocytes (100,000 cells/insert) and pericytes (30,000 cells/insert) were seeded onto the basal surface in BrainPhys Neuronal Medium supplemented with 2% NeuroCult SM1, 1% N2 Supplement-A, 20 ng/mL each of BDNF and GDNF, 1 mM dibutyryl-cAMP, and 200 nM ascorbic acid (Stemcell Technologies, Canada). Human brain microvascular endothelial cells were seeded onto the apical surface (30,000 cells/insert) in human endothelial serum-free medium supplemented with 0.5% B27, 20 ng/ml hbFGF (Thermo Fisher Scientific, USA), and 10 µM retinoic acid (Sigma-Aldrich, St. Louis, MO, USA). After 24 h, hbFGF and retinoic acid were withdrawn, and the models were incubated for another 24 h.

Subsequently, the BBB models were preconditioned with undiluted GBM patient plasma in the apical compartment for 24 h, followed by transendothelial electrical resistance (TEER) measurement using the CellZScope+ impedance spectrometer (nanoAnalytics, Münster, Germany). The Plasma was then renewed, 1 µM fadraciclib (in DMSO/PBS) was added apically, and the inserts were transferred to wells containing the corresponding patient-derived GBM cells in fresh co-culture medium. After 48 h, TEER and paracellular permeability to 4 kDa FITC–dextran (100 µg/ml, incubation time 1 h) were assessed. GBM cell viability was determined by Calcein-AM staining using a Spark^®^plate reader (TECAN). Endothelial ZO-1 (1:200, Thermo Fisher Scientific, USA) and astrocytic GFAP (1:100, Miltenyi Biotec, Germany) were analyzed by immunofluorescence using an Eclipse Ti microscope (Nikon, Tokyo, Japan) and the Spark plate reader.

### Spheroid invasion assay

Spheroids were generated by seeding 10,000 cells/well stably transduced to express the iRFP680 protein in a 96-well ULA-plate [27, 28]. All additional steps were done as described before [9].

### Spectral flow cytometry

Spectral flow cytometry was performed using three in-house multicolor panels as previously described [9, 29]. Panel 1 assessed proliferation and autophagy; panel 2, viability, methuosis, and immune regulation; and panel 3, immunogenic cell death, epithelial-mesenchymal transition (EMT), and stemness. Briefly, 0.2 × 10⁶ cells were stained with antibodies targeting extra- or intracellular antigens. All staining steps were performed using staining buffer (PBS, 2 mM EDTA, 2% BSA). The staining protocols for the three panels and the subsequent steps have been described previously [9, 29]. Measurements were performed using a 3 L-Cytek™ Aurora spectral flow cytometer. Data were analyzed using SpectroFlo™ Version 3.2.1. and FlowJo™ Version 10.6.1 (FlowJo, LLC; BD Becton Dickinson and Company, Franklin Lakes, NJ, USA).

### Immunofluorescence

Cells (5,000/well) were seeded in µ-Slide 18-well chambers (ibidi, Germany), allowed to adhere overnight, and treated for 2 × 72 h. After PBS washing, cells were fixed with 2% paraformaldehyde for 15 min, permeabilized with 0.5% Triton X-100 for 1 h, and blocked with 2% BSA for 1 h.

Primary antibodies, all diluted 1:50 in PBS containing 1% BSA and 0.5% Triton^®^ X-100, were incubated overnight and included Alexa Fluor^®^ 488 anti-ATF6α, Alexa Fluor^®^ 546 anti-PERK, Alexa Fluor^®^ 647 anti-IRE1α (all Santa Cruz Biotechnology), and Alexa Fluor^®^ 594 anti-phospho-H2A.X (Ser139) (BioLegend). Apotracker Green (BioLegend) was applied for 60 min. hMC3 microglia were stained for Connexin 43 (Alexa Fluor^®^ 488).

For CDK2 and CDK9 staining, mouse anti-human CDK2 (1A6; 1:25) and rabbit anti-human CDK9 (K.513.1; 1.50, both Thermo Fisher Scientific, overnight) were used. Primary antibodies were visualized with anti-mouse or anti-rabbit IgG with Alexa Fluor^®^ 647 conjugate (1:250, Biolegend) for 30 min. Cytoskeleton was stained with phalloidin green (1:50 in PBS, Biolegend) for 20 min.

Nuclei were counterstained with DAPI (Thermo Fisher Scientific). Images were acquired using a Zeiss Axiovert A1 microscope and analyzed with ImageJ (NIH).

### Visualization of intracellular organelles using ER-tracker, Lyso-Tracker, and Mito-Tracker

Cells were stained with MitoTracker^®^ Red CMXRos (20 nM; Cell Signaling Technology) and ER-Tracker™ Blue-White DPX (1 µM; Thermo Fisher Scientific) for 30 min at 37 °C. After PBS washing, LysoTracker™ Green DND-26 (50 nM; Thermo Fisher Scientific) was applied. Imaging was performed immediately using a Zeiss Axiovert A1 microscope.

### JC-10 assay

The mitochondrial membrane potential was evaluated using JC-10 assay (AAT Bioquest, Pleasanton, USA) according to the manufacturer’s instructions. Briefly, 5,000 cells/well were seeded into a 96-well plate and treated for 2 × 72 h with the respective agents. Cells were then washed once with prewarmed DPBS (w/o Ca2^+^/Mg) and incubated with 10 µM JC-10 solution in DMEM/F12 without supplements at 37 °C, 5% CO_2_ for 45 min. Fluorescence was measured at excitation/emission wavelengths of 490/525 nm and 540/590 nm using a Tecan Infinite 200 Pro plate reader. The ratio of fluorescence at 590 nm and 525 nm was calculated to assess the mitochondrial membrane potential.

### Measurement of the oxygen consumption rate and extracellular acidification rate using Seahorse

The oxygen consumption rate (OCR) and extracellular acidification rate (ECAR) were measured using a Seahorse XFe24 Extracellular Flux Analyzer (Agilent Technologies, Inc., Waldbronn, Germany) in accordance with the manufacturer’s instructions and as previously described [30, 31]. Briefly, 10,000 cells/well were seeded in Seahorse XFe24 plates and treated for 72 h with the respective agents. One hour before the measurement, the cells were washed, and the culture medium was replaced with XF Base Medium adjusted to pH 7.4 and supplemented with 10 mM glucose, 1 mM pyruvate, and 2 mM glutamine.

Mitochondrial respiratory function was assessed using the Seahorse XF Cell Mito Stress Test Kit (Agilent Technologies, Inc.). During the assay, oligomycin was injected through port A at a final concentration of 1.5 µM, followed by FCCP through port B at a final concentration of 1.5 µM and a combination of rotenone and antimycin A through port C, each at a final concentration of 0.5 µM. Basal respiration, ATP-linked respiration, spare respiratory capacity, proton leak, maximal respiration, and non-mitochondrial oxygen consumption were calculated using the Seahorse XF Cell Mito Stress Test Report Generator (Agilent Technologies, Inc.).

Following completion of the Seahorse measurement, cells were lysed in buffer containing 50 mM HEPES, 150 mM NaCl, 1 mM EDTA, 1% (v/v) Triton^®^ X-100, and 10% (v/v) glycerol. The protein content of each well was subsequently determined using the BCA Protein Assay Kit (Thermo Fisher Scientific), and absorbance was measured using a Spark^®^ Cyto 600 multimode microplate reader (Tecan Trading AG, Männedorf, Switzerland). OCR values were normalized to the protein content of the respective wells.

### Mitochondrial DNA (mtDNA) copy number analysis

The mtDNA copy number was analyzed using a multiplexed real-time qPCR (ViiA 7 Real-Time PCR System). After 2 × 72 h of treatment, GBM cells were harvested, stored at − 80 °C, and DNA was isolated using QIAamp DNA Mini Kit (Qiagen, Hilden, Germany). DNA (20 ng) was analyzed using TaqMan™ assays targeting mitochondrial ND1 (VIC-labeled; Hs02596873_s1) and nuclear RPPH1 (FAM-labeled; Hs03297761_s1). The mtDNA copy number was calculated as the ratio of ND1 Ct to RPPH1 Ct values.

### Tumor-conditioned medium

GBM cells were cultured in DMEM/F12 supplemented with 10% FCS, 1% penicillin/streptomycin, and 1 mM L-glutamine to ~ 80% confluency. After three PBS washes, cells were incubated in serum-free medium for 48 h. Supernatants were collected, centrifuged (1,000 × g, 10 min, 4 °C), sterile-filtered (0.22 μm), aliquoted, and stored at − 80 °C as tumor cell-conditioned medium (TCM).

### Semi-autologous co-culture of GBM cells and immune cells

The co-culture assay was done as described before [9, 32]. Immune phenotyping was done as follows: Cells were stained with FVD eFluor450 (1:250, 30 min, RT; BioLegend) for viability, followed by True-Stain Monocyte Blocker™ (BioLegend). Extracellular staining included (30 min, RT): BV421™ anti-human CD279 (PD-1) (BioLegend, San Diego, California, USA, 1:500), BV570™ anti-human CD8a (BioLegend, 1:160), BV750™ anti-human CD4 (BioLegend, 1:40), Spark Blue™ 550 anti-human CD3 (BioLegend, 1:100), PE anti-human CD25, REAfinity™ (Miltenyi Biotec, 1:200), BD Horizon™ APC-R700 Mouse Anti-Human CD127 (BD BioBiosciences, 1:200), FITC anti-human CD107a (LAMP-1) (BioLegend, 1:100), PE/Dazzle™ 594 anti-human CD152 (CTLA-4) (BioLegend, 1:50), AF^®^ 647 anti-human CD223 (LAG-3) (BioLegend, 1:50). After washing, samples were acquired on a Cytek Aurora flow cytometer and analyzed using SpectroFlo™ (v3.2.1) and FlowJo (v10.6.1).

### Co-culture invasion assay

Tumor spheroids were generated 3–4 days prior to embedding. Monocytes from healthy donor blood were isolated by density gradient centrifugation (Pancoll; PAN-Biotech), followed by CD14⁺ selection using MACS with CD14 antibodies (Miltenyi Biotec) according to the manufacturer’s instructions. For co-culture invasion assays, monocytes or hMC3 microglia were suspended in Matrigel containing the respective treatment and 1% EGF. Subsequent procedures were performed as described in the spheroid invasion assay protocol.

### Quantification of selected compounds in cell culture medium by LC-MS/MS

Cell culture supernatants were collected at indicated time points and centrifuged. Two aliquots were spiked with 2-isopropylmalic acid (Sigma-Aldrich) as internal standard, proteins were precipitated with LC-MS grade acetonitrile (Carl Roth), and samples were diluted prior to UHPLC–MS/MS analysis.

Chromatographic separation was performed on a Shimadzu Nexera series system coupled to a Shimadzu LCMS-8060 triple quadrupole mass spectrometer equipped with ESI source. Separation was achieved on a Shim-Pack GIST-HP PFPP column (2.1 × 150 mm, 3 μm). Data acquisition and processing were conducted using LabSolutions and LabSolutions Insight LCMS, respectively.

The “LC/MS/MS Method Package for Cell Culture Profiling Version 2” (Shimadzu) enabled gradient elution and MRM detection of 124 analytes within a 20-min run. Quantification was based on external calibration (≥ 6 levels; R² > 0.99) for each target compound; only signals with S/*N* > 10 were considered. Recovery was assessed using spiked culture medium.

### Multicellular 3D-bioprints

Patient-derived GBM cells, hAstro, and hMC3 were combined in a tricellular bioprinted co-culture model. Cells were labeled with CellTrace Far Red (GBM), CellTrace Yellow (HMC3), or CellTrace Violet (hAstro; all Thermo Fisher Scientific) according to the manufacturer’s protocol. Briefly, 1 × 10⁶ cells were stained (Yellow/Violet: 5 µM; Far Red: 1 µM, 20 min, 37 °C), diluted in medium, centrifuged, and mixed at a 3:1:1 ratio (GBM: hAstro: HMC3) in prewarmed 1.5% alginate/7.5% gelatin hydrogel (2 × 10⁶ cells/ml).

The bioink was loaded into a cartridge and printed at 22 °C using a GeSiM Bioscaffolder 3.3 (GeSiM, Germany) into poly-D-lysine-coated 12-well plates as grid-like scaffolds (250 μm nozzle, 50–80 kPa, 2 mm/s). Constructs were crosslinked with 5% microbial transglutaminase and 0.1 M CaCl₂ (10 min, RT) and cultured in 1:1:1 DMEM/F12:Astrocyte Medium: EMEM. Drug testing was performed after 5 days.

Viability and cell number were assessed using the ImageXpress Confocal HT.ai (Molecular Devices) and analyzed with MetaXpress 6 software.

### Isolation, analysis and spectral flow cytometry of extracellular vesicles

EVs were isolated from the supernatant of scaffolds collected before treatment and after day 7. The supernatant was diluted to 1 ml and centrifuged at 400 *x g* for 10 min. After centrifugation, 900 µl were transferred to new 1.5 ml tubes and centrifuged at 14,000 *x g* for 30 min. Subsequently, 800 µl of supernatant was removed. The remaining liquid was diluted to 1 ml and centrifuged again at 14,000 *x g* for 30 min. Afterwards, 900 µl were removed and the previous step was repeated. Finally, Evs were stored in the remaining 100 µl at -80 °C until analysis.

For quantification, Evs were diluted 1:10 in PBS and were analyzed regarding the particle concentration and size based on the nanoparticle tracking analysis using the NanoSight^®^ LM10 instrument (Malvern Instruments Ltd., Worcestershire, UK). Particle concentration from a PBS blank was subtracted from the sample concentration to exclude the external particles of the PBS as described [33].

For flow cytometric analysis, EV suspensions were adjusted to a concentration of 2.5 × 10^8^ particles. Membrane-enclosed Evs were identified by the retention of CellTrace signal and samples were stained with RY610-conjugated anti-human CD95 (Fas, clone DX2, stock 100 µg/ml, BD, 1:250 in filtered PBS, 30 min at room temperature). Measurements were performed using a Sony ID7000 flow cytometer. Evs were defined based on their fluorescence (CellTrace), and expression of CD95 (Fas) was analyzed within this population. To ensure specificity and reliability of EV detection, multiple controls were included: PBS controls with and without antibodies, Fas-unstained Evs, antibody titration controls, and serial dilutions to exclude swarm effects. Tumor cell and PBMC samples were used to verify marker localization. A detergent control was performed to confirm that detected signals originated from membrane-bound structures.

### Patient-derived organoids and autologous co-culture

Patient-derived organoids (PDOs) were established from eight patients using a fragment-based, architecture-preserving approach as previously described [30, 34]. Tumor tissue was washed with ice-cold DPBS, dissected into 0.5–1 mm pieces in Glioblastoma Dissection Medium (Hibernate A; Thermo Fisher Scientific) supplemented with 1% penicillin/streptomycin and 1% L-glutamine (both PAN-Biotech). After red blood cell lysis and washing, tissue pieces were cultured in GBO medium composed of DMEM/F12 and Neurobasal medium (1:1), MEM-NEAA (100×), human insulin, N2 (100×) and NCS21 (50×) supplements, penicillin/streptomycin, and L-glutamine in 12-well suspension plates. Cultures were maintained at 37 °C, 5% CO₂ on an orbital shaker (230 rpm) with regular medium changes. Viability was assessed using Calcein-AM (4 µM, 20 min) and imaged with a Zeiss LSM 780 with ZEN Black software v2.3 SP1. Additional viability analysis used the CellTiter-Glo 3D Kit, and luminescence was measured on a Tecan Infinite 200 Pro.

For autologous co-culture experiments, cryopreserved PBMCs from patient GBM41 were thawed by serial dilution in T-cell thawing medium (RPMI 1640, 1% penicillin/streptomycin, 1% L-glutamine, 10% fetal calf serum; all Pan-Biotech), followed by DNase treatment (37 °C, 15 min). PBMCs were resuspended at 2 × 10⁶ cells/ml in T-cell culture medium (RPMI 1640, 1% penicillin/streptomycin, 10% human serum, all Pan-Biotech; 1% GlutaMax-I, ThermoFisher Scientific; 150 U/ml IL-2, Immunotools, Friesoythe, Germany) and incubated overnight at 37 °C. PDOs were seeded into 96-well U-bottom plates the following day and either maintained in T-cell culture medium or embedded in Matrigel. PBMCs were harvested with PBS and added at 5 × 10⁵ cells/well together with DMSO or fadraciclib. Treatment was renewed after 72 h, and following a total of 2 × 72 h, Brefeldin A (BioLegend, 1:1,000) was added for 4 h. Supernatants were collected for further analysis. For Matrigel-embedded conditions, organoids and PBMCs were separated by liquefying the matrix at 4 °C for 30 min; PDOs were transferred to a new plate, and the PBMC-containing solution was diluted with pre-cooled PBS and centrifuged (300 × *g*, 4 °C, 5 min). For non-embedded co-cultures, PBMCs were harvested by resuspension in PBS. Flow cytometric analysis was performed as described above. PDO cytotoxicity was assessed by adding SYTOX™ Deep Red (Thermo Fisher Scientific, 1:1,000) in PBS and incubating at 37 °C for 30 min; after washing, fluorescence was measured on a Tecan Infinite 200 Pro at excitation/emission wavelengths of 640/680 nm.

### Chemokine and cytokine secretion profiles

Cytokines were evaluated in supernatants from 2D cultured cells and from PDOs cultured either alone or in co-culture with PBMCs using the LEGENDplex™ Human Proinflammatory Chemokine Panel 1 13-plex and Human CD8/NK Panel 13-plex (both from BioLegend). Supernatants were processed according to the manufacturer’s instruction. All measurements were performed on a spectral flow cytometer (3 L-Cytek™Aurora). Data were analyzed using SpectroFlo™ Version 3.2.1 and FlowJo™ Version 10.6.1, respectively. The BioLegend LEGENDplex™ Data Analysis Software Suite was used for statistical analysis.

### RNA sequencing and analysis

PDOs were treated for 2 × 72 h, and at least five PDOs per condition were pooled. Total RNA was isolated using RNeasy Mini Kit (Qiagen, Venlo, Netherlands) with DNase treatment according to the manufacturer’s instruction. RNA quality was verified by Nanodrop measurement, and samples with OD_260/280_ ≥ 2.0 and OD_260/230_ ≥ 2.0 were used for RNA sequencing, which was done with polyA enrichment, performed on the Illumina PE150 sequencing platform with 30 Mio reads/sample (paired end analysis). Bioinformatic analysis included differentially expressed genes (DEG) between control and fadraciclib (defined by a |log₂ fold change| > 1 and adjusted p-value < 0.05). Gene Ontology (GO) enrichment analyses were performed using the web tool DAVID [3, 4, 35, 36]. Volcano and bar plots were created in R studio (version 4.4.2), and STRING networks were visualized using cytoscape (version 3.10.3).

### Statistics

Statistical analysis and data visualization were performed using Prism GraphPad 10.6.0 (GraphPad Software, San Diego, CA, USA; RRID: SCR_002798). Each data set was tested for normality with the Shapiro-Wilk test. All statistical tests and sample sizes are reported in the figure legends. Data are presented as mean ± SD, with individual values given in the plots. The criterion for significance was set to *p* < 0.05.

## Results

### CDKs are highly expressed in primary GBM and patient-derived models, and dual CDK2/9 inhibition reduces GBM cell viability

**Fig. 1:**
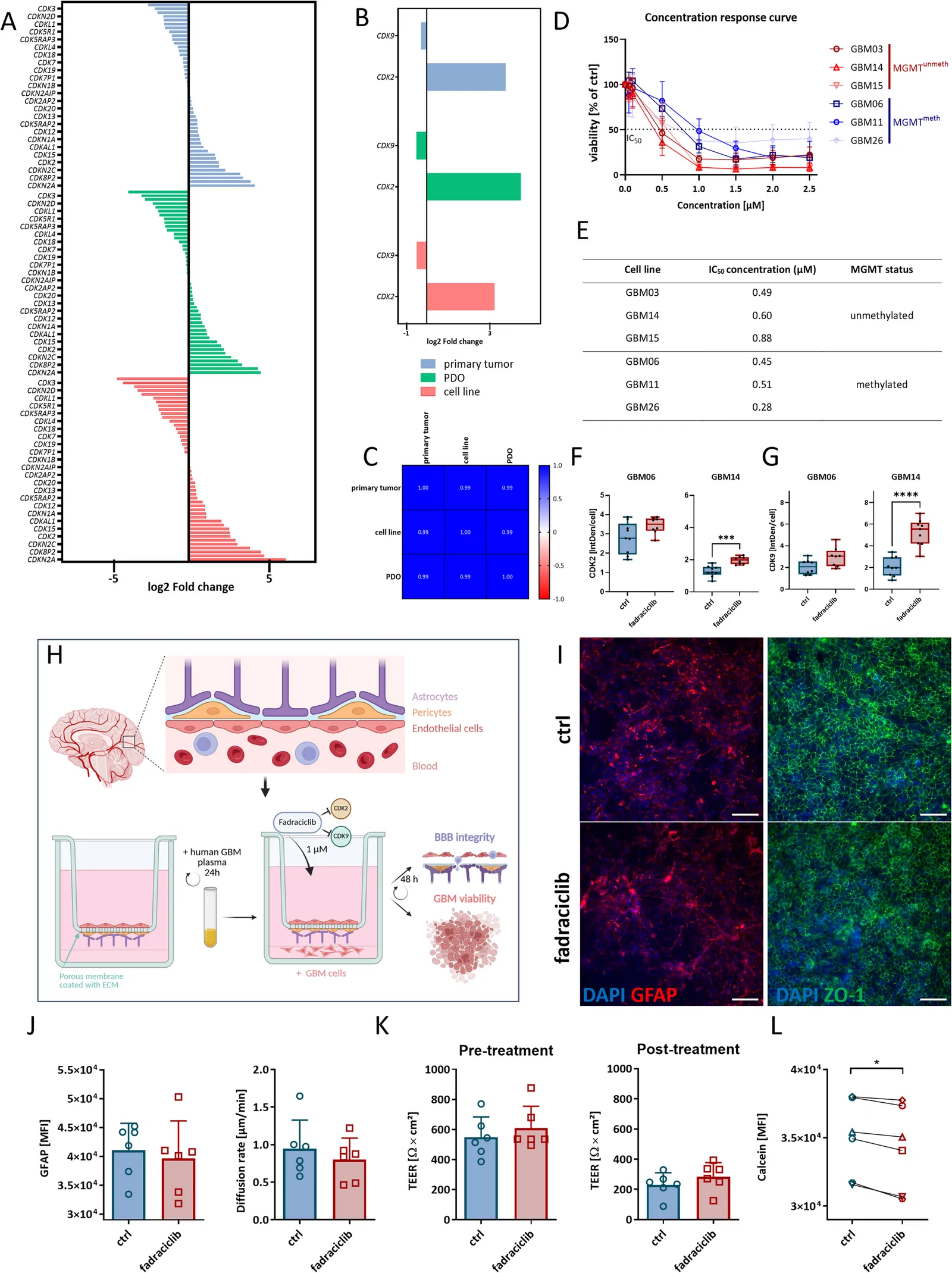
Characterization of CDK expression and analysis of treatment response. **A** Waterfall plots showing different CDK expression profiles in primary GBM, patient-derived organoids, and patient-derived cell lines. RNA bulk sequencing included *n* = 7 primary GBM cases, *n* = 12 patient-derived cell lines, and *n* = 3 patient-derived organoids (PDO). **A** The expression profiles of *n* = 48 individual genes were analyzed and the log2 fold change in relation to brain cortex is shown. **B** Expression profile of CDK2 and CDK9 within different sample types reveal overexpression of CDK2 across samples (log2 fold change relative to brain cortex). **C** Correlation analysis was done using Pearson correlation coefficient for every pair of data. **D**, **E** Concentration-dependent viability and invasiveness reduction after fadraciclib treatment. Six GBM cell lines with (un-)methylated MGMT status were treated for 2 × 72 h with escalating concentrations of fadraciclib. Viability was assessed using crystal violet staining, and results were used to calculate IC_50_ values. *n* = 3–4, mean ± SD. **F**, **G** Signal intensity of CDK2 (**F**) and CDK9 (**G**) immunofluorescence staining after 2 × 72 h fadraciclib treatment (0.5 µM) was quantified as integrated density relative to the amount of cells. *n* = 3. Mann-Whitney test. *** *p* < 0.001, **** *p* < 0.0001. **H**-**M** Impact of fadraciclib on spheroid invasion and induction of cell stress. **H**-**L** Blood-brain barrier model. **H** Experimental workflow of the triple-culture BBB model. Brain microvascular endothelial cell (BMEC)-like cells, astrocytes, and pericytes were cocultured on Matrigel-coated TC inserts, with astrocytes and pericytes seeded basolaterally followed by BMEC-like cells on the apical side. Autologous plasma from GBM patients was then added, followed by fadraciclib (1 µM) or vehicle control (DMSO). Inserts were transferred to 24-well plates containing 2D-cultured GBM cells and incubated for 48 h. TEER and GBM cell viability were assessed as readouts of BBB integrity and drug penetration, respectively. Created in BioRender. Kaps, C. (2026) https://BioRender.com/5jy2686 (**I**) Shown are representative immunofluorescence images of astrocytes (GFAP) and tight junctions (ZO-1) to demonstrate the preservation of the barrier integrity after 48 h of treatment with DMSO (control) or fadraciclib (**J**) The barrier integrity was quantified by analyzing the mean fluorescence intensity (MFI) of GFAP from the immunofluorescence stainings with a TECAN Spark reader (left) and evaluation of the diffusion rate of FITC-dextran after treatment (right). *n* = 6 different patient-derived GBM cell lines, mean + SD. Wilcoxon test. **K** TEER measurements pre- and post-treatment completed the testing of BBB integrity, where no interruption of integrity was detectable. *n* = 6 different patient-derived GBM cell lines, mean + SD. Wilcoxon test. **L** The viability of GBM cells was evaluated by measurement of the Calcein AM intensity after 48 h treatment. *n* = 6 different patient-derived GBM cell lines, mean + SD. Wilcoxon test, * *p* < 0.05

Analysis of primary GBM samples identified *CDK2* among the top ten expressed genes, along with *CDKN2A* and *CDKN2C* (Fig. 1A). This pattern was preserved in PDOs and early-passage cell lines (≤ P10), showing strong correlations between sample types (*r* = 0.99–1.0; Fig. 1B, C). To evaluate the effect of CDK2/9 inhibition, the CDK inhibitor fadraciclib was selected, which selectively inhibits CDK2 and CDK9 as previously characterized [12, 37]. Patient-derived GBM cell lines with both MGMT-methylated and -unmethylated status were treated with escalating concentrations of fadraciclib, over two 72-h cycles (Fig. 1D). All lines exhibited concentration-dependent sensitivity in the nanomolar range, independent of *MGMT* status, with GBM26 being most sensitive (IC₅₀ = 0.28 µM; Fig. 1E). As higher concentrations (> 1 µM) did not increase efficacy, 0.5 µM was used for subsequent experiments.

Despite reduced cell viability following treatment, immunofluorescence analysis revealed increased protein abundance of CDK2 (Fig. 1F, Supplementary Fig. 1A) and CDK9 (Fig. 1G, Supplementary Fig. 1B) upon fadraciclib exposure, consistent with a compensatory response. This effect reached statistical significance in GBM14 (CDK2: *p* < 0.001; CDK9: *p* < 0.001).

### Dual CK2/9 inhibitor fadraciclib penetrates the blood-brain barrier while retaining anti-glioblastoma activity

A major limitation for central nervous system-targeted therapeutics is restricted BBB access, which excludes most small molecules from reaching intracranial tumors at therapeutically relevant concentrations. To determine whether fadraciclib overcomes this barrier, we employed a triple-culture BBB model incorporating autologous patient plasma, which minimizes nonspecific protein-binding effects and provides physiological preconditioning representative of the GBM patient environment prior to drug treatment (Fig. 1H–L). Following 48 h of fadraciclib treatment, we first verified that barrier integrity was preserved under drug exposure, since compromised tight junctions could produce false-positive penetration through paracellular leakage rather than active or passive transcellular transport. Immunofluorescence staining of astrocytes and tight junction markers showed no evidence of barrier disruption, a finding corroborated by unchanged GFAP signal intensity and stable FITC-dextran diffusion rates across the endothelial layer (Fig. 1I, J). Consistent with this, TEER measurements—a functional readout of paracellular permeability—showed no significant reduction in transendothelial electrical resistance following fadraciclib exposure (Fig. 1K), together confirming that the barrier remained functionally intact throughout treatment.

Having established that BBB integrity was maintained, we next asked whether fadraciclib nonetheless crossed the barrier in a biologically active form. Using an indirect transwell assay, in which GBM cells were exposed only to fadraciclib that had diffused across the intact endothelial-astrocyte barrier, we observed a significant reduction in tumor cell viability (Fig. 1L). This result formally demonstrates that fadraciclib not only crosses an intact BBB but retains sufficient antitumoral potency to impair GBM cell survival on the abluminal side—a critical prerequisite for its consideration as a central nervous system-penetrant therapeutic candidate.

### Dual CDK2/9 inhibition reduces spheroid invasion and is associated with cellular stress in patient-derived GBM cells

**Fig. 2:**
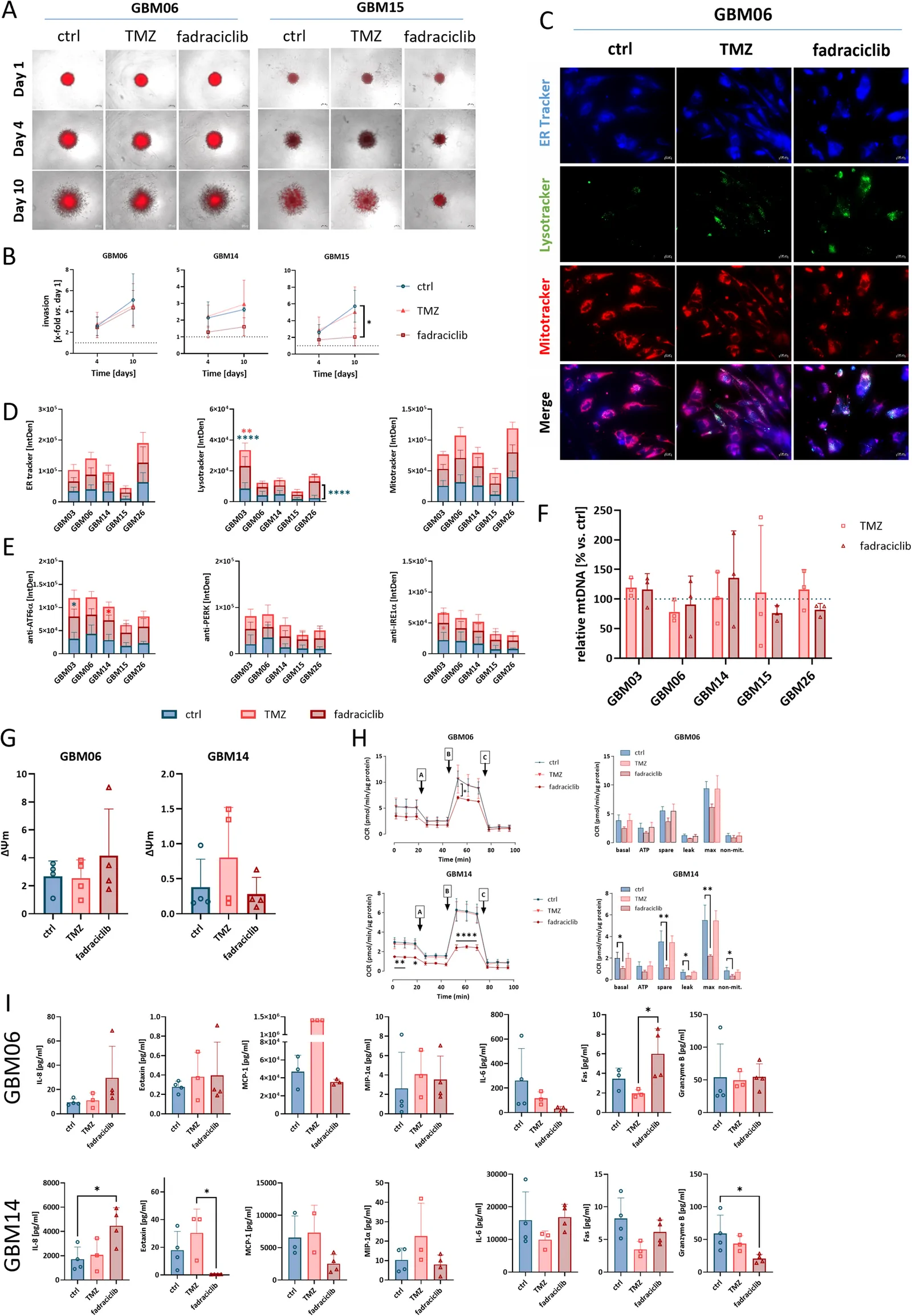
CDK2/9 inhibition triggers cell death-associated pathways and alters chemokine secretion profiles in GBM cells. **A** Representative images of GBM spheroids, which were embedded in Matrigel and treated with TMZ (10 µM), fadraciclib (0.5 µM), or DMSO as a control for 2 × 72 h. Invasiveness was monitored on days 1, 4, and 10 using a fluorescence microscope. **B** The invasiveness was quantified relative to day 1 using the NIR-fluorescent signal. *n* = 4, mean ± SD. Two-way ANOVA (Sidak’s multiple comparison test). **C**-**F** Impact of treatment on cell stress and mitochondrial integrity. (**C**) Shown are representative images of GBM06, which were treated for 2 × 72 h with TMZ (10 µM), fadraciclib (0.5 µM), or DMSO. Cells were afterwards stained with ER-, Lyso-, and Mitotracker to visualize cell organelles. **D** Signal intensity of each staining was quantified. *n* = 4, mean ± SD. Two-way ANOVA (Tukey’s multiple comparison test); ** *p* < 0.01; **** *p* < 0.0001 vs. ctrl. **E** GBM cells were stained for ER stress markers ATF6α, PERK, and IRE1α and quantified. *n* = 4, mean ± SD. Two-way ANOVA (Tukey’s multiple comparison test); * *p* < 0.05. **F** Mitochondrial DNA content was assessed via qPCR. *n* = 3, mean ± SD, each dot represents one biological replicate. **G** JC-10 Mitochondrial Membrane Potential Assay. *n* = 3, mean ± SD, unpaired t-test. **H** Oxygen consumption rate (OCR) of GBM06 and GBM14 cells measured by Seahorse XFe24 Extracellular Flux Analysis following a mitochondrial stress test protocol with sequential injection of oligomycin (1.5 µM), FCCP (1.5 µM), and antimycin A/rotenone (0.5 µM each). Left panels show OCR kinetics over time; right panels display derived parameters including basal respiration (basal), ATP-linked respiration (ATP), spare respiratory capacity (spare), proton leak (leak), maximal respiration (maximal), and non-mitochondrial oxygen consumption (non-mit.). *n* = 3–4, mean ± SD, one-way ANOVA (Tukey’s multiple comparison test); *p < 0.05, **p < 0.01, ****p < 0.0001 vs. control. **I** Chemokine and cytokine profiles from supernatants of GBM06 and GBM14 cells. *n* = 3–4, mean ± SD. One-way ANOVA (Tukey’s multiple comparison test) or Kruskall-Wallis test (Dunn’s multiple comparisons test), * *p* < 0.05

In 3D spheroid assays, fadraciclib significantly reduced GBM invasiveness, particularly in GBM15 (*p <* 0.05 vs. control; Fig. 2A, B). Treated spheroids remained compact, indicating suppressed invasion, whereas temozolomide (TMZ) had no effect, highlighting the specific anti-invasive activity of fadraciclib.

Then, we examined the cellular stress response in the endoplasmic reticulum (ER), lysosomes, and mitochondria after two consecutive 72-h treatments with fadraciclib, TMZ, or control using 2D-cultured GBM cells (Fig. 2C-I). Both fadraciclib and TMZ increased fluorescence in lysosomes, ER, and mitochondria versus control, indicating stress responses in all GBM lines, though the effect was generally lower with fadraciclib. GBM03 and GBM26 exhibited particularly strong lysosomal staining, suggesting cell line-specific susceptibility (Fig. 2C, D). Fadraciclib treatment also elevated UPR markers PERK, ATF6α, and IRE1α (Fig. 2E), but this was not unique to CDK2/9 inhibition, as TMZ produced similar UPR activation, with GBM15 showing minimal changes. Increased MitoTracker signal prompted assessment of mtDNA, which remained unchanged, indicating that the signal reflects mitochondrial swelling rather than biogenesis (Fig. 2F). Effects on the mitochondrial level were further investigated by assessing the mitochondrial membrane potential (ΔΨm) and the mitochondrial respiratory function (Fig. 2G, H). When assessing the ΔΨm via JC-10 assay, GBM06 showed an overall higher mitochondrial membrane potential compared to GBM14. Interestingly, fadraciclib treatment lead to a hyperpolarization in GBM06 (4.16 (fadraciclib) vs. 2.69 (DMSO)), but not in GBM14 (0.28 (fadraciclib) vs. 0.38 (DMSO)). In contrast, TMZ had the opposite effects with no impact on the ΔΨm in GBM06 (2.54 (fadraciclib) vs. 2.69 (DMSO)) and a hyperpolarization in GBM14 (0.80 (fadraciclib) vs. 0.38 (DMSO)). Measurements obtained using the Seahorse Extracellular Flux XFe24 analyzer confirmed that fadraciclib treatment induced a marked impairment of mitochondrial bioenergetic function in GBM cells, as reflected by reduced basal respiration, ATP production, spare respiratory capacity, and proton leak (Fig. 2H).

Moreover, chemokine and cytokine secretion profiles revealed induction of a proinflammatory stress response, with slight differences between the two cell lines (Fig. 2I). In GBM06, fadraciclib induced increased release of IL-8 and Fas, together with reduced IL-6 secretion. In contrast, GBM14 exhibited a broad suppression of secreted factors, including eotaxin, MCP-1, Fas, and Granzyme B, suggesting that CDK2/9 inhibition engages distinct context-dependent biological programs across GBM.

### Dual CDK2/9 inhibition triggers cell death- and stress-associated pathways in patient-derived GBM cells and is associated with metabolic alterations

**Fig. 3:**
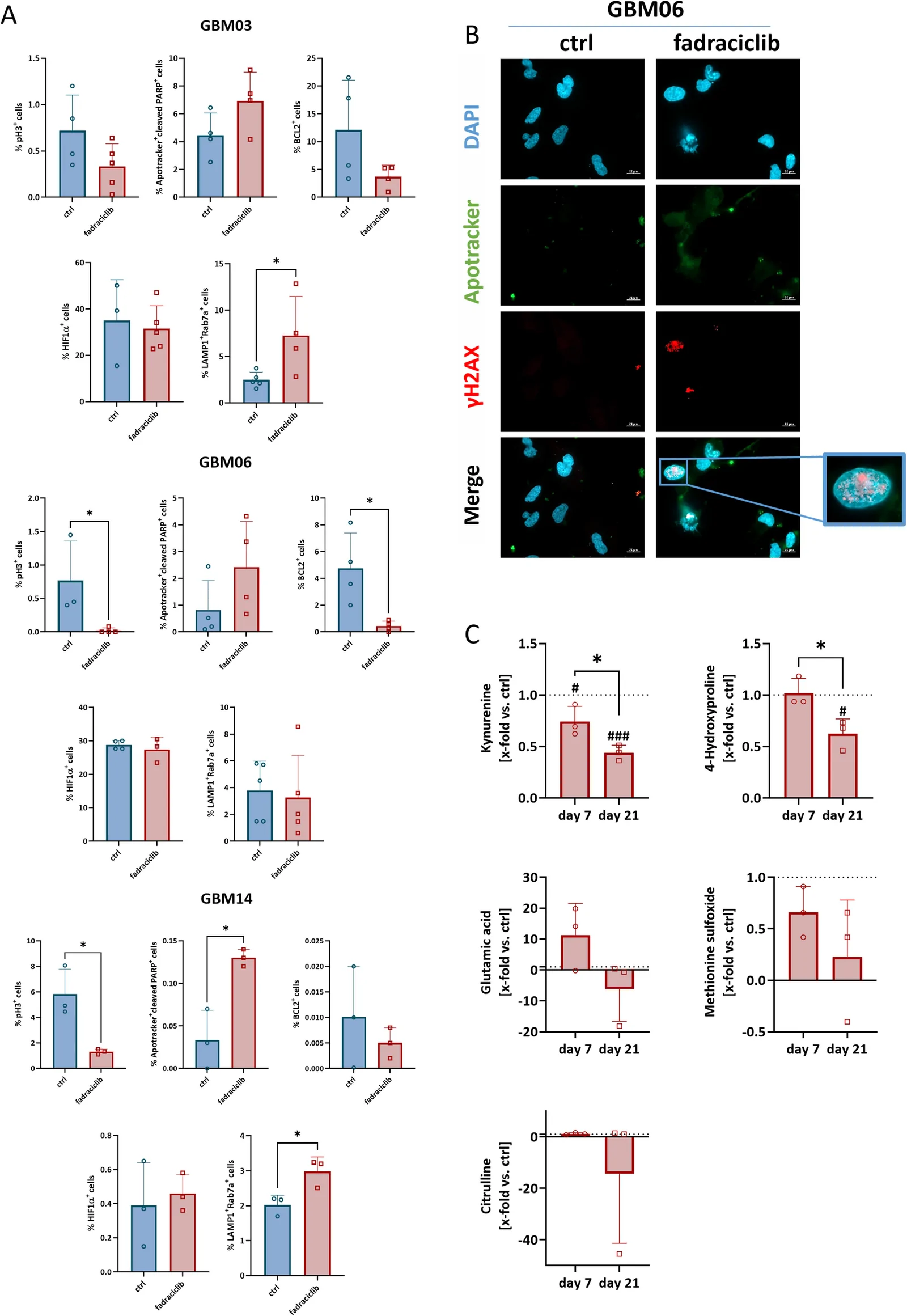
CDK2/9 inhibition induces metabolic rewiring in GBM cells. **A** GBM cells (GBM03, GBM06, GBM14) were treated for 2 × 72 h with DMSO or fadraciclib. Effects of the treatment were analyzed using spectral flow cytometry. *n* = 3–5, mean + SD. Unpaired t-test; * *p* < 0.05. **B** Cells were stained for apoptosis (Apotracker) and DNA damage (γH2AX) markers after treatment. Shown are representative images of GBM06. *n* = 2. **C** The secretion profiles of key metabolites relevant to GBM migration and metabolism were analyzed in cell-free supernatant obtained from GBM14 cells cultured as 3D spheroids and subjected to treatment with fadraciclib for either 2 or six treatment cycles. *n* = 3, mean + SD. Unpaired t-test; * *p* < 0.05; # *p* < 0.05 vs. day 7

Fadraciclib-induced viability loss in patient-derived GBM cells was associated with activation of cell death pathways (Fig. 3). All lines showed similar deregulation of cell death markers, independent of MGMT methylation (Fig. 3A). Reduced pH3-positive cells, particularly in GBM06 and GBM14, indicated cell cycle arrest and decreased mitotic activity. Concurrently, apoptotic markers—including Apotracker and cleaved PARP—were elevated, while BCL2-positive cells decreased (*p <* 0.05 vs. control), confirming apoptosis. GBM03 and GBM14 also showed significant increases in LAMP1- and Rab7a-positive cells (*p <* 0.05), suggesting enhanced autophagy. Immunofluorescence revealed elevated γH2AX foci, indicating DNA damage induction (Fig. 3B).

The secretion of key metabolites relevant to GBM migration and metabolism was then analyzed in cell-free supernatants from GBM14 spheroids after two (= day 7) or six (= day 21) treatment cycles (Fig. 3C).

Fadraciclib markedly reduced kynurenine, a tryptophan catabolite, at both time points. Levels of 4-hydroxyproline, a marker of collagen turnover and a critical component of extracellular matrix (ECM) remodeling, were also significantly reduced, suggesting a potentially impaired invasive potential. Other metabolic byproducts, including methionine sulfoxide and citrulline, declined with prolonged treatment, consistent with broader metabolic alterations induced by fadraciclib.

Overall, these findings show that fadraciclib induces broad metabolic alterations in GBM cells, while reducing invasiveness and secretion of immunosuppressive metabolites.

### CDK2/9 blockade modulates immunomodulatory features of GBM cells

**Fig. 4:**
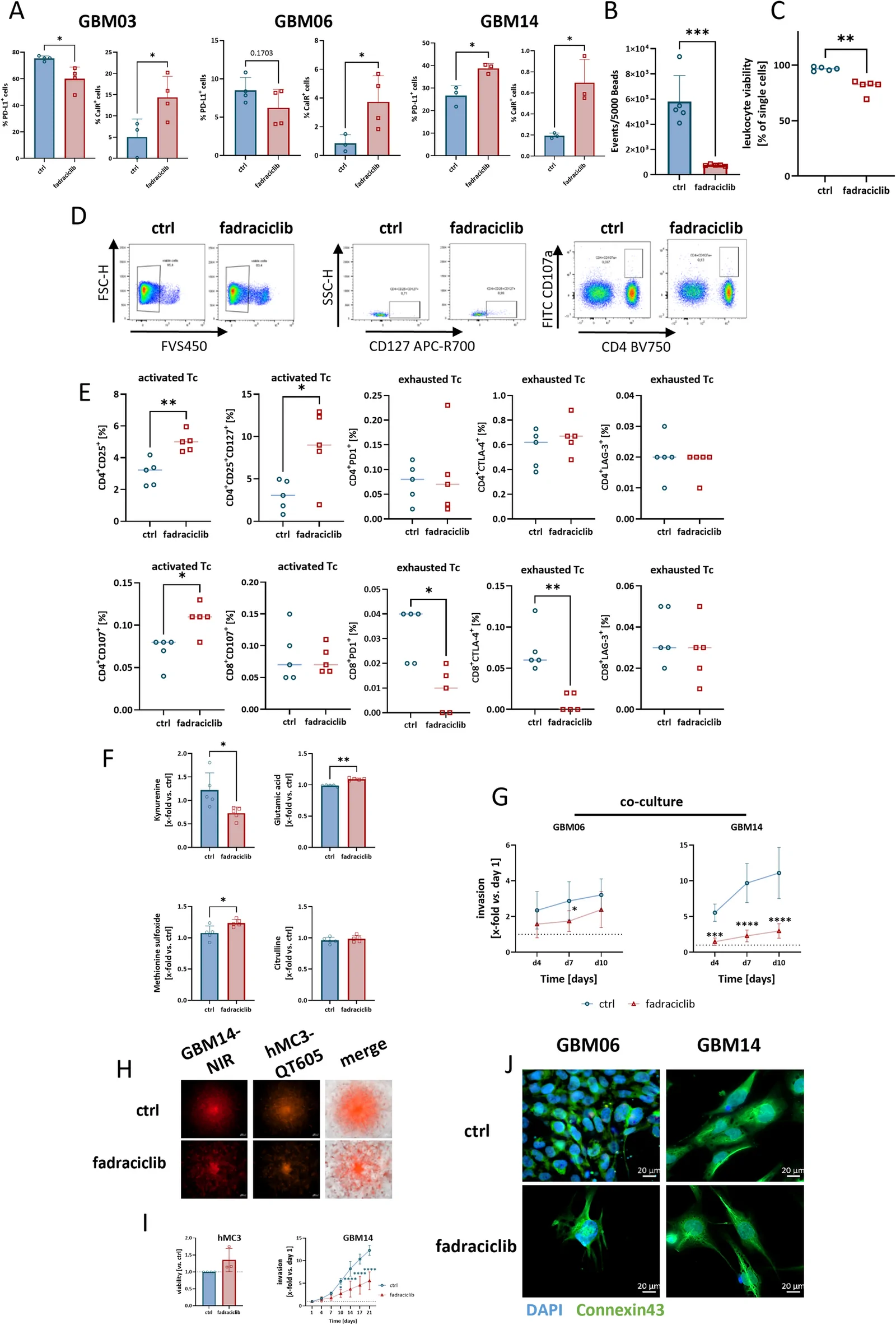
CDK2/9 inhibition mediates immune modulation and alters invasion in myeloid-GBM co-cultures. **A** GBM cells (GBM03, GBM06, GBM14) were treated for 2 × 72 h with DMSO or fadraciclib. Treatment effects were analyzed using spectral flow cytometry. *n* = 3–4, mean + SD. Unpaired t-test; * *p* < 0.05. (B-E) Semi-autologous co-culture of GBM14 cells and peripheral blood mononuclear cells (PBMC) +/- fadraciclib (0.5 µM). **B** The number of residual cells was quantified. *n* = 5 individual donors, mean + SD. Unpaired t-test; *** *p* < 0.001. **C**-**E** Spectral flow cytometry to determine (**C**) viability and (**D**, **E**) phenotype of PBMCs after co-culture with GBM14 cells +/- fadraciclib (0.5 µM). **C** Viability of PBMCs. *n* = 5 individual donors; horizontal line within scatter plots indicates the median. Unpaired t-test; ** *p* < 0.01. **D** Representative flow cytometry dot plots. (**E**) Phenotype of PBMCs. Horizontal line within scatter plots indicates the median. Unpaired t-test; * *p* < 0.05; ** *p* < 0.01. **F** The secretion profiles of key metabolites relevant to GBM migration and metabolism were analyzed in the cell-free supernatant obtained from GBM14 cells co-cultured with PBMCs and subjected to treatment with fadraciclib for two treatment cycles. *n* = 5, mean + SD. Unpaired t-test; * *p* < 0.05; ** *p* < 0.01. **G**-**I** GBM cells were embedded in Matrigel containing (**G**) peripheral CD14^+^ monocytes or (**H**, **I**) hMC3 cells. The co-culture was treated for 2 × 72 h or a total of six cycles for 21 days with DMSO or fadraciclib. Invasion was monitored using a fluorescence microscope and quantified as the relative change in fluorescence intensity compared to day 1. *n* = 4, mean ± SD, Two-way ANOVA (Tukey’s multiple comparison test); * *p* < 0.05; *** *p* < 0.001; **** *p* < 0.0001. **J** Exposure to tumor-conditioned medium (either obtained from GBM06 or GBM14 cells) and assessment of Connexin 43 abundance in hMC3 microglia +/- fadraciclib (0.5 µM). Representative images are shown, *n* = 2

Then, we analyzed immunoregulatory molecules on GBM cells (Fig. 4). In GBM03, which had high basal PD-L1 (> 70%), fadraciclib treatment significantly reduced PD-L1 levels (*p <* 0.05 vs. control). GBM06, with low baseline PD-L1 (< 10%), also showed decreased PD-L1 in residual cells, while GBM14 displayed a modest increase (*p* < 0.05 vs. ctrl). Importantly, all cell lines demonstrated significant calreticulin surface translocation following treatment (*p <* 0.05 vs. control, Fig. 4A), indicating engagement of an ER stress-linked immunoregulatory axis. Three distinct GBM-immune cell co-culture systems were then employed (Fig. 4B-H): (I) 2D co-culture with peripheral blood mononuclear cells (PBMCs), (II) 3D spheroids with peripheral monocytes, and (III) 3D spheroids with tissue-resident microglia. In the first setting, fadraciclib markedly reduced residual tumor cells (*p <* 0.001 vs. control; Fig. 4B), with only modest effects on PBMC viability (~ 80% vs. >95% in controls, Fig. 4C). Immune profiling revealed increased activated CD4⁺ non-regulatory T cells (CD4⁺CD25⁺, CD4⁺CD25⁺CD127⁺, Fig. 4D, E) and cytotoxic degranulation (CD107a^+^), without induction of T cell exhaustion in either CD4^+^ nor CD8^+^ T cells (Fig. 4D, E). Metabolite analysis in cell-free supernatants supported these findings: Kynurenine levels were significantly lower in fadraciclib-treated samples, along with higher levels of methionine sulfoxide (Fig. 4F).

In the second approach, the impact of fadraciclib on GBM invasiveness was studied in the presence of peripheral monocytes, the predominant myeloid subset in primary GBM (Fig. 4G). In GBM06, monocyte co-culture did not enhance invasiveness (5.1 ± 2.5-fold vs. 3.2 ± 0.9-fold), while GBM14 displayed significantly higher invasiveness (2.6 ± 0.1-fold vs. 11.1 ± 3.6-fold). Fadraciclib consistently suppressed invasion, with GBM14 showing significant reductions at all time points (Fig. 4G). Finally, in the third model, microglia—specialized, tissue-resident macrophages of the central nervous system that are frequently co-opted by tumor cells to exploit intrinsic immunosuppressive pathways and facilitate immune evasion [38] — were co-cultured with 3D-spheroids (Fig. 4H, I). Fadraciclib did not affect microglial viability, but maintained strong anti-invasive effects, significantly inhibiting tumor invasion over 21 days (Fig. 4I, right).

Collectively, fadraciclib not only modulates GBM–immune cell interactions, but also reduces invasiveness in clinically relevant GBM-immune co-culture models.

### Dual CDK2/9 inhibition shapes the phenotype of activated microglia

We next examined microglia to assess fadraciclib`s immune modulatory effects. Tumor-naïve hMC3 cells exposed to tumor-conditioned medium from GBM06 or GBM14 cells upregulated the gap junction protein Connexin 43 (Fig. 4J), indicating tumor-driven alterations in microglial gap junction protein level. While functional intercellular communication was not directly assessed, Connexin 43 upregulation may also reflect early microglial remodeling in response to tumor-derived signals.

### Antitumoral effects of dual CDK2/9 inhibition are preserved in sophisticated 3D models and independent of MGMT promoter methylation status

**Fig. 5:**
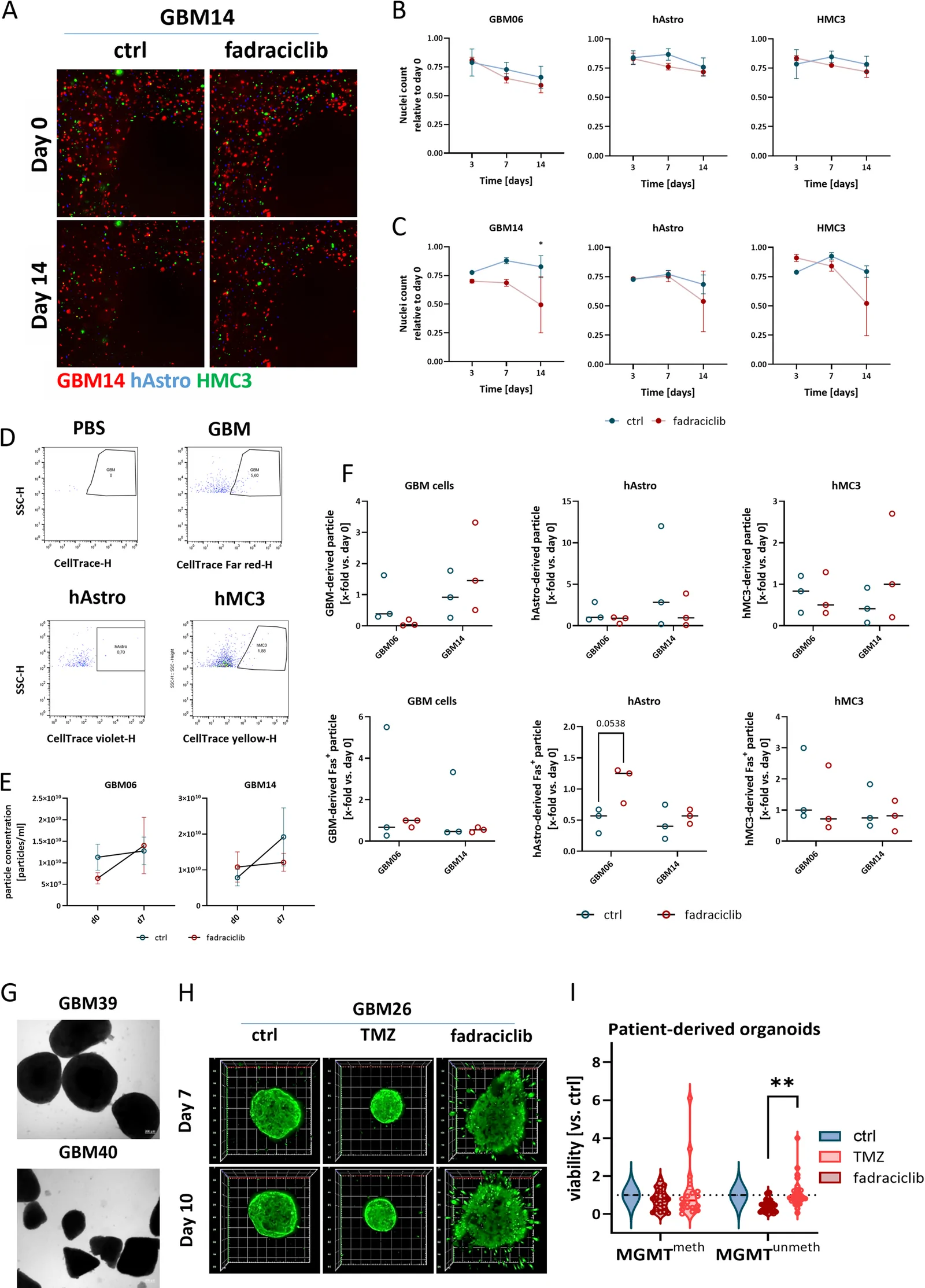
Fadraciclib demonstrates strong therapeutic potential in biomimetic multicellular systems and alters to release of extracellular vesicles. **A**-**C** Multicellular 3D-bioprints. **A** Representative images taken on the ImageXpress Confocal HT.ai and analyzed with MetaXpress 6 software. **B**, **C** Longitudinal assessment of cell viability. Cells were labeled with CellTrace Far Red (GBM), CellTrace Yellow (hMC3), or CellTrace Violet (hAstro) and printed as described in material and methods. Viability of fadraciclib-treated and control scaffolds was analyzed over a period of 10 days. Given is the number of nuclei counts relative to day 0 (= start of treatment). *n* = 1–3/timepoint, mean ± SD. Two-way ANOVA (Sidak’s multiple comparison test); * *p* < 0.05. **D**-**F** Extracellular vesicle profiling. **D** Representative flow cytometric scatter plots showing particle measurement stratified by cell type, according to the specific CellTrace signal. PBS was used as negative control. **E** Absolute particle concentration in supernatants of scaffolds at different time points (day 0, day 7). *n* = 3, mean ± SD. **F** Flow cytometric particle quantification. Data show the number of measured particles/µl at day 7 relative to day 0 stratified by cell type (i.e. GBM cells, hAstro, or hMC3, upper) and the number of cell-derived CD95^+^ particles. *n* = 3, mean ± SD. Two-way ANOVA (Tukey’s multiple comparison test). **G**-**I** PDO approach. **G** Representative light microscopic images of individual PDOs from GBM39 and GBM40 at week 4 after tumor resection are shown. **H**, **I** PDOs were treated for 2 × 72 h with DMSO, TMZ, or fadraciclib. Viability was monitored using Calcein AM staining and confocal laser scanning microscopy on day 7 and 10 after treatment started. **H** Shown are merged Z-stack images of GBM26 (LSM780). **I** The viability of PDOs was quantified using the CellTiter 3D Glo assay after a total of 10 days. *n* = 3–4 individual PDOs/GBM case. Horizontal line within violin plots indicates the median. Two-way ANOVA (Sidak’s multiple comparison test); ** *p* < 0.01

A tricellular bioprinted co-culture model comprising GBM cells (GBM06 or GBM14), human astrocytes (hAstro) and microglia (HMC3) was employed to further investigate the effects of fadraciclib in a multicellular context (Fig. 5A-C). Cells were labeled with distinct CellTrace dyes and monitored over 14 days using a high-content imaging system, with fadraciclib administered in two 72-h treatment cycles. Consistent with our previous findings, GBM06 showed no noteworthy response towards fadraciclib, as reflected by stable nuclei counts relative to day 0 (Fig. 5B). Notably, non-malignant cells remained likewise unaffected by fadraciclib in this co-culture system. In contrast, co-cultures containing the more sensitive cell line GBM14 displayed a reduction in total cell numbers, reaching statistical significance by day 14 (0.83 vs. 0.49 relative to day 0, p < *0.05*, Fig. 5C). While hAstro were not affected by fadraciclib, HMC3 showed lower cell counts in GBM14 co-cultures following fadraciclib treatment (0.79 vs. 0.52 relative to day 0, *p* = 0.088). In addition, EV profiles from cell-free supernatants were analyzed (Fig. 5D-F). Although total EV numbers remained stable over time, subtle changes in EV composition and phenotypic signatures were observed between control and treated conditions (Fig. 5F). Notably, in hAstro-derived EVs from GBM06-containing bioprints, a trend towards elevated Fas levels was detected, which may reflect reactive processes within the scaffolds and is in accordance with the increased Fas concentration already found in the supernatant (Fig. 2I). However, this interpretation remains exploratory given the limited sample size and warrants further investigation. Moreover, this effect was absent in hAstro-EVs from GBM14 bioprints.

**Fig. 6:**
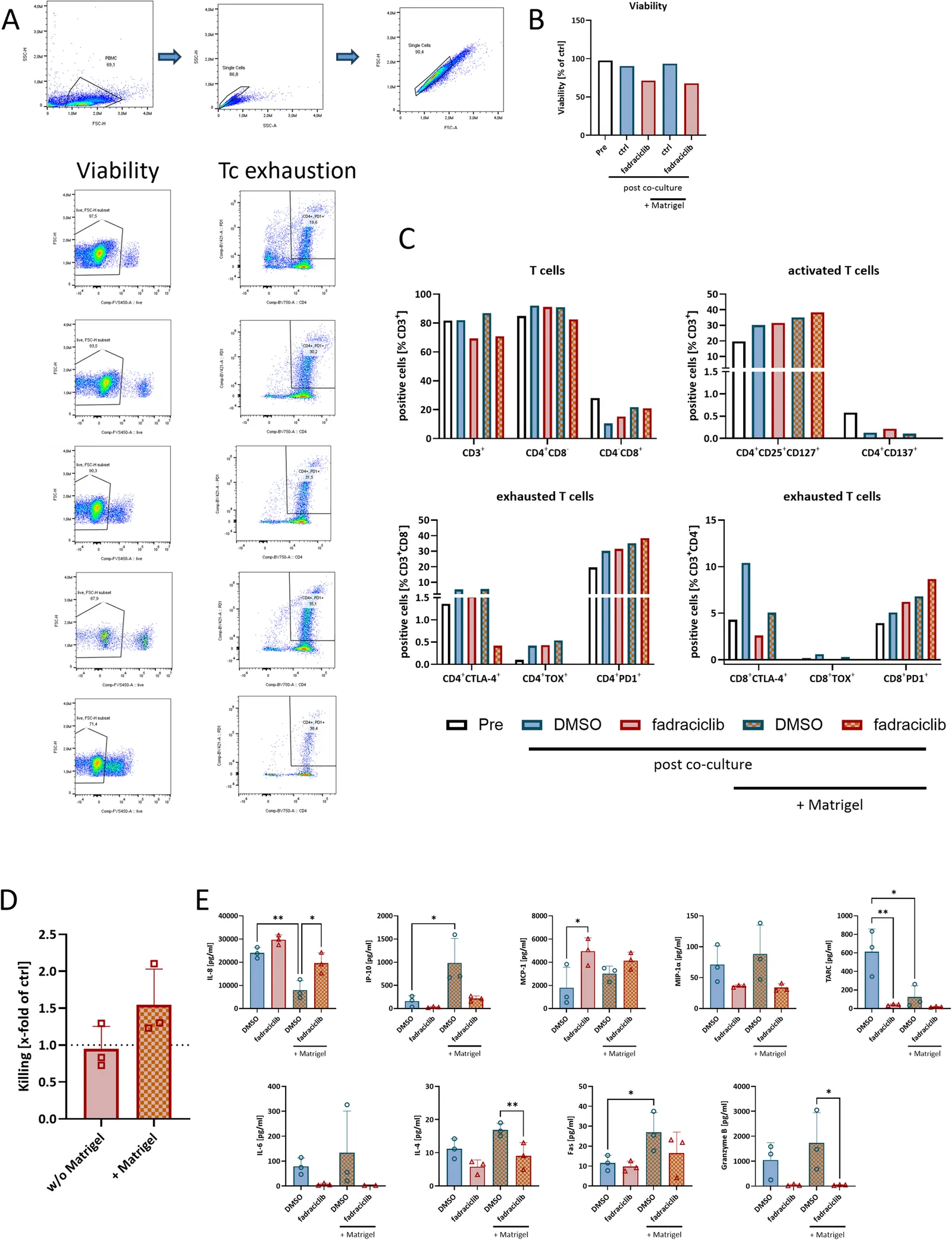
Antitumoral effects of CDK2/9 blockade in autologous PDO-immune cell co-cultures. **A**-**E** Autologous co-culture of GBM41 PDOs and peripheral blood mononuclear cells (PBMC) +/- fadraciclib (0.5 µM), using two different culture conditions, i.e. with or without Matrigel. **A**-**C** Flow cytometric phenotyping. **A** Gating strategy and representative flow cytometry dot plots. **B** Viability of immune cells before and after co-culture. **C** Phenotype of PBMCs before and after co-culture. **B**, **C** *n* = 1 experiment. **D** Viability of PDOs after co-culture, normalized to controls (without fadraciclib). *n* = 3 individual PDOs. mean ± SD. **E** Chemokine and cytokine profiles from supernatants. *n* = 3 individual PDOs. mean ± SD. One-way ANOVA (Tukey’s multiple comparisons test). * *p* < 0.05; ** *p* < 0.01

To confirm this further, patient-derived organoids (PDOs) from eight GBM cases (exemplary light microscopic imaging shown in Fig. 5G) were generated to validate the viability reduction observed in 2D and 3D models. PDOs were treated with fadraciclib or TMZ, using a 2 × 72-h regimen with a four-day follow-up. While control- and TMZ-treated organoids retained spheroidal morphology, fadraciclib-treated PDOs showed marked structural changes, disintegrating into smaller clusters or single cells (Fig. 5H). CellTiter-Glo 3D assays confirmed that fadraciclib significantly reduced PDO viability to 77.2% ± 12.4 (MGMT-methylated) and 43.2% ± 6.2 (MGMT-unmethylated), relative to untreated controls (Fig. 5I). In contrast, TMZ had no effect on PDO viability (116.8% ± 35.0 and 112.1% ± 14.9, respectively). Hence, fadraciclib outperformed the standard-of-care agent TMZ in both molecular subtypes, including MGMT-methylated PDOs, underscoring its superior efficacy in in preclinical multicellular GBM models. To further investigate fadraciclib’s immunomodulatory potential, PDOs from GBM41 were co-cultured with autologous PBMCs, either floating or embedded in Matrigel (Fig. 6). Flow cytometric analysis (Fig. 6A-C) revealed a comparable reduction of viable PBMCs under fadraciclib treatment in both settings (71.4% without Matrigel vs. 67.9% with Matrigel). Focusing on the T cell compartment, the CD3⁺ fraction was modestly reduced upon fadraciclib treatment, with no proportional change in the CD4⁺CD8⁻ subpopulation. While the CD4⁻CD8⁺ fraction was generally lower compared to pre-co-culture conditions, Matrigel-embedded PDOs were associated with a higher proportion of these cells, independent of treatment. An activated, non-regulatory CD4⁺CD25⁺CD127⁺ T cell population was maintained across all conditions. Notably, fadraciclib reduced CTLA-4-expressing T cells (CD4⁺ or CD8⁺), with more pronounced effects in the ECM-embedded setting. TOX⁺ T cell populations remained consistently low across all conditions, and PD1 expression was comparable between treatment groups, collectively supporting an activated rather than exhausted T cell phenotype. Tumor cell killing, assessed by SYTOX assay (Fig. 6D), was higher in the Matrigel-embedded condition (0.95 vs. 1.54 relative to control). Cytokine profiling further underscored distinct differences between the two culture conditions, which were further modulated by fadraciclib treatment (Fig. 6E). Whereas IL-8 and MCP-1 levels increased following treatment, several other immunomodulatory, partly immunosuppressive mediators, including IP-10 (CXCL10), MIP-1α, TARC, and IL-4, were markedly reduced. Notably, Fas and Granzyme B were also decreased in this complex autologous co-culture setting, demonstrating that CDK2/9 inhibition induces a selective and context-dependent remodeling of the secretory milieu.

### Fadraciclib reprograms immune-associated pathways and promotes a proneural shift in GBM patient-derived organoids

**Fig. 7:**
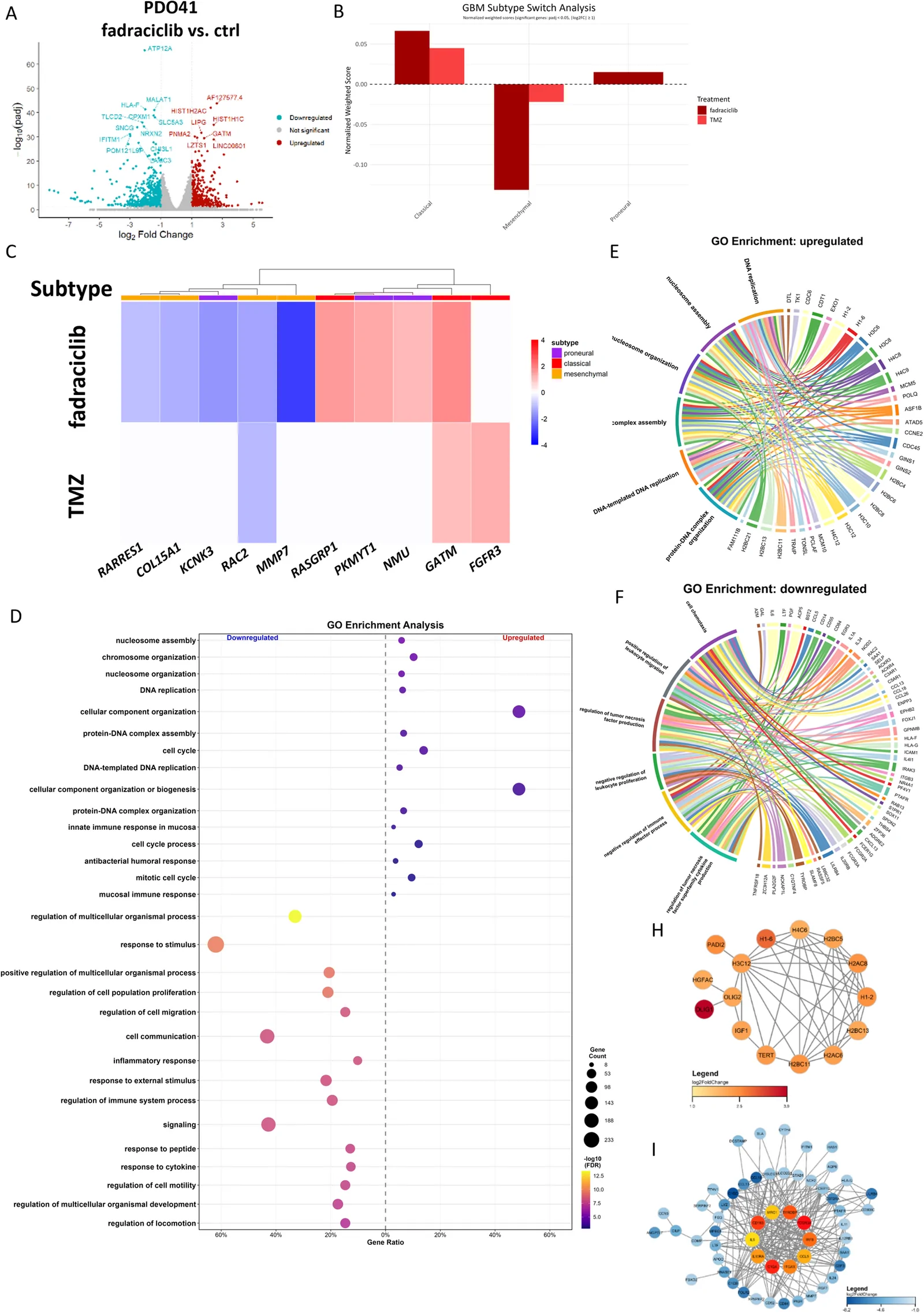
Fadraciclib induces a molecular switch in GBM cells. **A**-**I** RNA bulk sequencing approach. **A** Volcano plot showing significantly deregulated genes in PDO41 after fadraciclib treatment (|log₂ fold change| > 1 and padj < 0.05). **B**, **C** GBM subtype switch analysis reveals transcriptional changes associated with a molecular shift after fadraciclib treatment. **B** Bar plot and (**C**) heatmap showing singular genes triggering molecular switch in residual GBM cells. **D** Modulation of immune cell-associated process. PDOs of GBM41 were treated for 2 × 72 h with DMSO or fadraciclib. Bubble plot showing biological process mostly affected by the treatment identified using gene ontology (GO) enrichment analysis (left: downregulation; right: upregulation). **E**, **F** Chord diagram to visualize the relationships between genes and pathways. **G**, **H** STRING network analysis was performed to visualize the interaction of deregulated proteins based on the deregulated DEGs. The ten proteins with the most interactions were additionally highlighted for the downregulated proteins

To gain initial insight into the molecular mechanisms underlying fadraciclib response, transcriptomic profiling was performed in PDO41, selected as an exemplary GBM case for this single-case study (Fig. 7). Bulk RNA sequencing identified 1080 differentially expressed genes (DEGs), with 459 upregulated and 621 downregulated (Fig. 7A). Tumor-promoting genes such as *MALAT1*,* SNCG*,* POM121L9*, and *CHI3L1* were significantly downregulated, whereas genes associated with chromatin regulation (*HIST1H2AC* and *HIST1H1C*), and the tumor suppressor *LZTS1* were upregulated, suggesting chromatin remodeling and tumor inhibition.

Subtype-specific analysis revealed a fadraciclib-induced shift from a mesenchymal to a hybrid classical/proneural transcriptional profile in PDO41, an effect not seen with TMZ (Fig. 7B, C). *MMP7* was significantly downregulated, while *PKMYT1* and *GATM* were upregulated.

Gene ontology (GO) enrichment analysis of DEGs highlighted that upregulated genes were enriched in nucleosome assembly, chromosome organization, DNA replication, and other processes related to cell cycle progression and chromatin remodeling (Fig. 7D, E, Supplementary Fig. 2A-C). Downregulated genes were enriched for immune response, cell communication, and migration, as well as inflammatory signaling and proliferation (Fig. 7D, F). Overall, GO analysis underscores fadraciclib’s dual impact on chromatin regulation and immunomodulatory pathways, linking transcriptional reprogramming with reduced invasiveness and altered immune signaling (Supplementary Fig. 2A-C).

To investigate the functional relevance of transcriptional changes, STRING analysis was performed on the top 100 up- and downregulated genes, revealing distinct protein–protein interaction networks (Fig. 7H, I). The upregulated network (15 proteins) was enriched for histone variants (e.g., H1-6, H2AC8, PADI2) involved in chromatin remodeling, along with transcriptional regulators such as OLIG1/2 and TERT (Fig. 7H; Table 2). The downregulated network (55 proteins) comprised immune-related factors involved in cytokine signaling and macrophage polarization (Fig. 7I; Table 3). Maximal clique centrality (MCC) identified key hub proteins, including IL6, IL10RA, CCL5, IRF8, CD163, MRC1, ITGAX, FCGR3A, C1QA, and TYROBP. Fadraciclib also modulated LncRNA expression (Supplementary Fig. 2D), upregulating oncogenic LncRNAs (AF127577.4, LINC00601, LINC002481) and downregulating others (e.g., LINC02078, AC127496.5, AC087667.1), highlighting a complex layer of transcriptional regulation.

**Table 2:** Network-associated upregulated proteins in GBM41 PDOs following fadraciclib treatment and their relationship to CDK2/9

Protein	Group	Relation to CDK2/9?	Source
H1-2	Linker histones	H1 histones are phosphorylated by both CDK2 and CDK9 as part of the P-TEFb; CDK2 interacts with molecules like DOT1L, LSD1, MSL1 which enable histone modification, CDK9 plays a role in the ubiquitination of H2	PMID: 28539972; PMID: 32494624; PMID: 19575011
H1-6			
H2AC6	Core histones		
H2AC8			
H2BC5			
H2BC11			
H2BC13			
H3C12			
H4C6			
OLIG1	Transcription factors	OLIG2 represses CDK2 inhibitor p21^WAF1/CIP1^	PMID: 17296553
OLIG2			
HGFAC	Growth factors	IGF1 activates CDK2 by repressing p27^Kip1^; HGFAC activates HGF and HGF induces pathway that phosphorylates CDK2 allowing cell cycle progression	PMID: 15033176; PMID: 26148348
IGF1			
PADI2	Enzymes	PADI2 facilitates the interaction of RNA polymerase II and P-TEFb via citrullination of RNA polymerase II; TERT promotes degradation of CDK inhibitor p21	PMID: 30472187; PMID: 39329419
TERT			

**Table 3:** Network-associated downregulated proteins in GBM41 PDOs following fadraciclib treatment and their relationship to CDK2/9

Protein	Group	Relation to CDK2/9?	Source
C1QA	Immune Response, inflammation & TME	Silencing of FOLR2 in NSCLC is associated with an upregulation of p21 and p27; IRF8 induce p21 and resulted in accumulation of p27 in NSCLC; IL6 induces STAT3 which associated with CDK9 to build an active complex; IL24 downregulates miR-221, thereby upregulating p27; G-CSF (CSF3) activates JAK-STAT signaling resulting in stimulation of the expression of p27^Kip1^	PMID: 30415267; PMID: 31594449; PMID: 17956865; PMID: 27940575; PMID: 27789332
C1QB			
C1QC			
FCER1G			
FCGR3A			
TYROBP			
CD163			
CD300C			
FOLR2			
MRC1			
SIGLEC1			
STAB1			
ITGAX			
LILRB4			
CD52			
CD84			
HLA-G			
SLA			
IRF8			
IL10RA			
IL12RB1			
IL6			
IL11			
IL24			
CSF2RA			
CSF3			
FGF7			
CCL5			
CCL13			
CCL18			
PF4V1			
FGG	Coagulation & hemostasis	PTAFR binds platlet-activating factor which showed to upregulate p21	PMID: 25950475; PMID: 28157211
PTAFR			
SERPINF2			
HAS1	ECM	CCN5 overexpression is associated with downregulation of CDK2 in OSCC	PMID: 31889370
MMP7			
COMP			
CCN5			
DCSTAMP			
CILP			
SLCO2B1	Lipid & small molecule transport	-	-
PIGR			
APOD			
CYTH4			
ANGPTL7	Angiogenesis & vascular	AQP9 overexpression is associated with an upregulation of p21 in CRC	PMID: 28640255
AQP9			
LYZ	Antimicrobial & defence	LTF induces p21^Cip1^ and p27^Kip1^ levels, thereby inhibiting CDK2; CDK2 levels were reduced in pancreatic cancer cells due to IFITM1 silencing	PMID: 35563638; PMID: 32434425
LTF			
RNASE1			
MPEG1			
NCF2			
IFITM1			
SAA1	Acute phase response	-	-
XPNPEP2	Metabolic & enzymatic	FBXO2 degrades WEE1 which acts as an inhibitor of CDK2 via phosphorylation	PMID: 40676478; PMID: 29175378
FBXO2			

Together, these exploratory finding suggest that fadraciclib may impair immune-related pathways while promoting a hybrid classical–proneural phenotype, coupled with transcriptional reprogramming involving chromatin organization, cell cycle progression, and post-transcriptional regulation. These findings provide a basis for future transcriptomic studies across a larger PDO cohort.

## Discussion

In this study, we confirm the cellular, molecular, and immuno-modulatory effects of dual CDK2/9 blockade in preclinical GBM models. Fadraciclib disrupts both cell cycle progression and transcriptional regulation, and has shown promise in preclinical [13, 39, 40] and clinical studies (NCT04983810) across multiple malignancies, including GBM. However, its potential impact on immune regulation and cellular crosstalk remains incompletely understood. This gap is particularly relevant given increasing recognition of the brain’s immune microenvironment as a key driver of tumor progression and therapy resistance [41]. The reported IC₅₀ values obtained across different primary GBM cell lines with different MGMT promoter methylation status fall within the clinically relevant range and compare favorably to other clinically approved CDK inhibitors [42, 43]. Notably, we demonstrate for the first time that fadraciclib crosses an in vitro BBB triple-culture model while retaining antitumoral efficacy against GBM cells, a key prerequisite for effective systemic therapy against GBM and other malignancies of the central nervous system. Preserved barrier integrity, confirmed independently by diffusion and TEER measurements, indicates that the observed reduction in tumor cell viability reflects genuine drug penetration rather than nonspecific barrier disruption. Beyond its cytotoxic effect, fadraciclib also impaired spheroid invasion, suggesting a link to CDK9/P-TEFb-mediated transcriptional control of genes involved in cell migration and invasion. This aligns with findings by Shan et al., who showed that CDK9 promotes MMP9 expression in lung cancer cells—a protease frequently overexpressed in GBM known to facilitate extracellular matrix degradation and invasiveness [44, 45]. Hence, CDK9 inhibition by fadraciclib may suppress MMP9 expression, contributing to the observed anti-invasive phenotype.

Fadraciclib sensitivity was associated with global cellular stress, including ER alterations, increased lysosomal and mitochondrial abundance, as well as impairment of mitochondrial bioenergetic function. Importantly, fadraciclib also increased γH2AX foci, consistent with an enhanced DNA damage response that may contribute to cell death. This, in turn, may promote the release of tumor-associated antigens and potentially enhance immune recognition of GBM cells—an important consideration given the profoundly immunosuppressive tumor microenvironment (TME) of GBM [46]. Additionally, fadraciclib triggered metabolic rewiring, promoting a shift toward glycolytic metabolism. This metabolic reprogramming was accompanied by reduced invasiveness and decreased immunosuppressive characteristics, suggesting that fadraciclib may help remodel the TME into a less aggressive and more immune-permissive state.

While the antitumor effects of CDK2/9 inhibition are well documented [12, 40, 47], immunomodulatory effects have not been previously reported. Using different co-culture models, we present novel evidence for the immuno-modulatory, pro-inflammatory effects of dual CDK2/9 blockade. Fadraciclib reduced PD-L1 expression in two of three cell lines - a notable contrast to CDK4/6 inhibitors - which often stabilize PD-L1. This suppression was accompanied by increased secretion of IL-8 and Fas, decreased viability and invasiveness of both tumor and myeloid cells, and upregulation of Connexin 43 in tissue-resident microglia, suggesting enhanced intercellular communication within an inflammatory milieu. These data indicate that CDK2/9 inhibition triggers a pro-inflammatory yet stress-adapted state, in which treatment-induced immune activation coexists with compensatory programs. Furthermore, fadraciclib modulated the phenotype of peripheral leukocytes in co-culture with GBM cells, promoting T cell activation and enhancing cytotoxicity. At the same time, the context-dependent reshaping of the secretory milieu indicates that this response is not uniformly stimulatory, but rather characterized by concurrent attenuation of selected chemotactic and cytotoxic signals. These findings support a multifaceted immunomodulatory role for CDK2/9 inhibition, engaging both innate and adaptive immunity. Consistent with this, prior work has shown that dinaciclib—a broader CDK inhibitor—induces histone-dependent immunogenic cell death in pancreatic cancer [48, 49].

Notably, the antitumor and immunomodulatory effects of fadraciclib were retained across both MGMT promoter–methylated and –unmethylated GBM subtypes in translational PDO models. In these sophisticated translational systems, fadraciclib outperformed TMZ, suggesting its potential to prevent or overcome chemotherapy resistance [50]. Using a multicellular 3D bioprinted system of malignant and non-malignant cells, our data further support the potential therapeutic efficacy of fadraciclib. Non-malignant cells were not affected by CDK2/9 blockade, suggesting a favorable safety profile within the highly sensitive brain microenvironment. However, responses differed between cell lines: GBM14 exhibited a marked reduction in viability, whereas GBM06 remained largely insensitive. Although the precise mechanisms underlying sensitivity and resistance remain unclear, our observation of elevated levels of Fas on astrocyte-derived extracellular vesicles may indicate pro-inflammatory, apoptotic, or activation-related signaling driven by reactive astrocytes [51].

At the transcriptional level, these effects were associated with broad disruption of gene expression, affecting both oncogenic and tumor-suppressive pathways. Fadraciclib downregulated tumor-promoting lncRNAs such as *MALAT1* and *POM121L9* [52–54], while upregulating anti-tumoral genes like *LZTS1*, which has been shown to inhibit PI3K/Akt signaling and suppress proliferation in hepatocellular carcinoma [55–57]. Additionally, lncRNAs such as AP003108.3 and LINC02078—known to modulate TGF-β signaling *via* miRNA sponging in colorectal cancer [58] – were significantly downregulated following treatment, further supporting a role for fadraciclib in reprogramming tumor-promoting transcriptional networks.

We also observed increased expression of histone-associated genes, suggesting enhanced chromatin remodeling and compaction as a downstream effect of transcriptional inhibition. Chromatin remodeling may reduce DNA accessibility, driving tumor cells into a quiescent, proneural-like state characterized by reduced aggressiveness but potential resistance to cell cycle–targeted therapies. Alternatively, histones, released extracellularly under oxidative stress, can act as danger-associated molecular patterns, promoting anti-tumor immunity. Prior studies have identified extracellular histones as key mediators of immunogenic cell death [49, 50]. Targeting these quiescent, transcriptionally reprogrammed—and potentially more immunogenic—phenotypes could improve treatment efficacy by promoting selective elimination of vulnerable cell clones. Thus, combination strategies aimed at exploiting this immunogenic shift may offer a promising therapeutic avenue, independent of the specific resistance mechanisms employed by individual clones.

Finally, despite the inclusion of several advanced human-relevant new approach methodologies, the present study is limited by the absence of in vivo validation. The spheroid, bioprinting co-culture, and PDO-based systems used here provide mechanistic insight into invasion-associated phenotypes and treatment-related immune responses. Still, they do not fully capture the complexity of the native brain tumor microenvironment, necessitating validation in appropriate in vivo models to determine whether these effects are preserved in the context of brain invasion and tumor-associated immune compartments.

## Conclusion

This study provides novel evidence that dual CDK2/9 inhibition extends beyond canonical cell cycle control to include pivotal roles in modulating immune cell biology. The compound’s dual mechanism—simultaneously attenuating tumor-supportive immune responses and inducing chromatin-driven transcriptional reprogramming—likely contributes to its antitumor efficacy in GBM. Nevertheless, further investigation is warranted to elucidate the precise molecular pathways involved across GBM subtypes and to develop rational combination therapies that enhance fadraciclib’s therapeutic potential.

## Supplementary Information

Supplementary Material 1: Supplementary Figure 1: Representative images of immunofluorescence staining of CDK2 (A) and CDK9 (B) after 2 x 72h treatment with fadraciclib (0.5 µM) or DMSO (ctrl).

Supplementary Material 2: Supplementary Figure 2: Visualization of the most significantly affected LncRNAs and pathways after fadraciclib treatment. (A-C) Altered pathways include inflammatory response, cell proliferation, and immune signaling. Overall, the most downregulated GO terms clustered into immune regulation, cell migration (A, B), and intracellular signaling (C), further corroborating an immunomodulatory effect of fadraciclib. Visualization was done using the software Cytoscape. (D) Volcano plot showing the expression profile of LncRNAs (log₂ fold change).

Supplementary Material 3: Supplementary Figure 3: Gating strategy. Spectral flow cytometry was conducted after autologous co-culture of GBM PDOs and peripheral immune cells. Thereafter, immune cells were harvested, counted and stained with a panel fo antibodies as described in material and methods. The hierarchical gating strategy is shown for detecting T cells and their functional exhaustion status.

## Data Availability

Raw and processed RNAseq data is accessable via the NCBI Gene Expression Omnibus (GEO) under the accession number GSE326468. All relevant data are also available from the corresponding authors upon request. Cortex tissue was used as reference for healthy tissue and the data used for the analyses described in this manuscript were obtained from the GTEx Portal and the GTEx Analysis V10.

## Abbreviations

BSA: Bovine serum albumin
CDK: Cyclin-dependent kinase
CDKi: Cyclin-dependent kinase inhibitor
DEG: Differentially expressed genes
ECM: Extracellular matrix
EMT: Epithelial mesenchymal transition
ER: Endoplasmic reticulum
EV: Extracellular vesicles
GBM: Glioblastoma
GO: Gene ontology
MCC: Maximal clique centrality
MGMT: O6-methylguanine-DNA methyltransferase
mtDNA: Mitochondrial DNA
PBMC: Peripheral blood mononuclear cells
PDO: Patient-derived organoid
P-TEFb: Positive transcription elongation factor b
RNAPII: RNA polymerase II
TCM: Tumor cell-conditioned medium
TME: Tumor microenvironment
TMZ: Temozolomide
